# Cheilitis Granulomatosa in Childhood: Unveiling an Uncommon Cause of Lip Enlargement

**DOI:** 10.1002/ccr3.70246

**Published:** 2025-02-27

**Authors:** Shintaro Fujiwara, Yousuke Higuchi, Yuma Nishimura, Sayuri Yokomizo, Yoko Shinno

**Affiliations:** ^1^ Department of Pediatrics NHO Okayama Medical Center Okayama Japan; ^2^ Department of Dermatology NHO Okayama Medical Center Okayama Japan; ^3^ Department of Pathology NHO Okayama Medical Center Okayama Japan

**Keywords:** cheilitis granulomatosa, dermatology, granulomatous cheilitis, lip swelling, pediatric

## Abstract

Cheilitis granulomatosa, a rare condition in children, is marked by painless, sudden lip swelling, which can last for an extended period. Since cheilitis granulomatosa can precede gastrointestinal symptoms in some cases of Crohn's disease, a comprehensive evaluation before administering systemic inflammatory modulatory treatments and long‐term follow‐up is necessary.

## 1 Case Presentation

**FIGURE 1:**
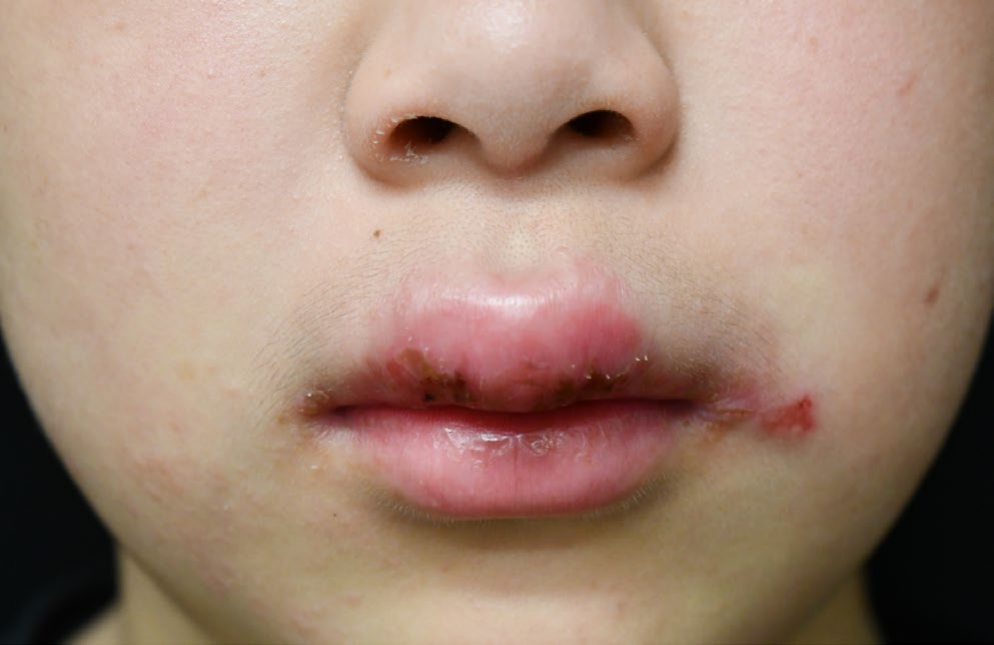
Angular cheilitis and painless lip swelling of both lips, especially the upper lip with erosion.

**FIGURE 2:**
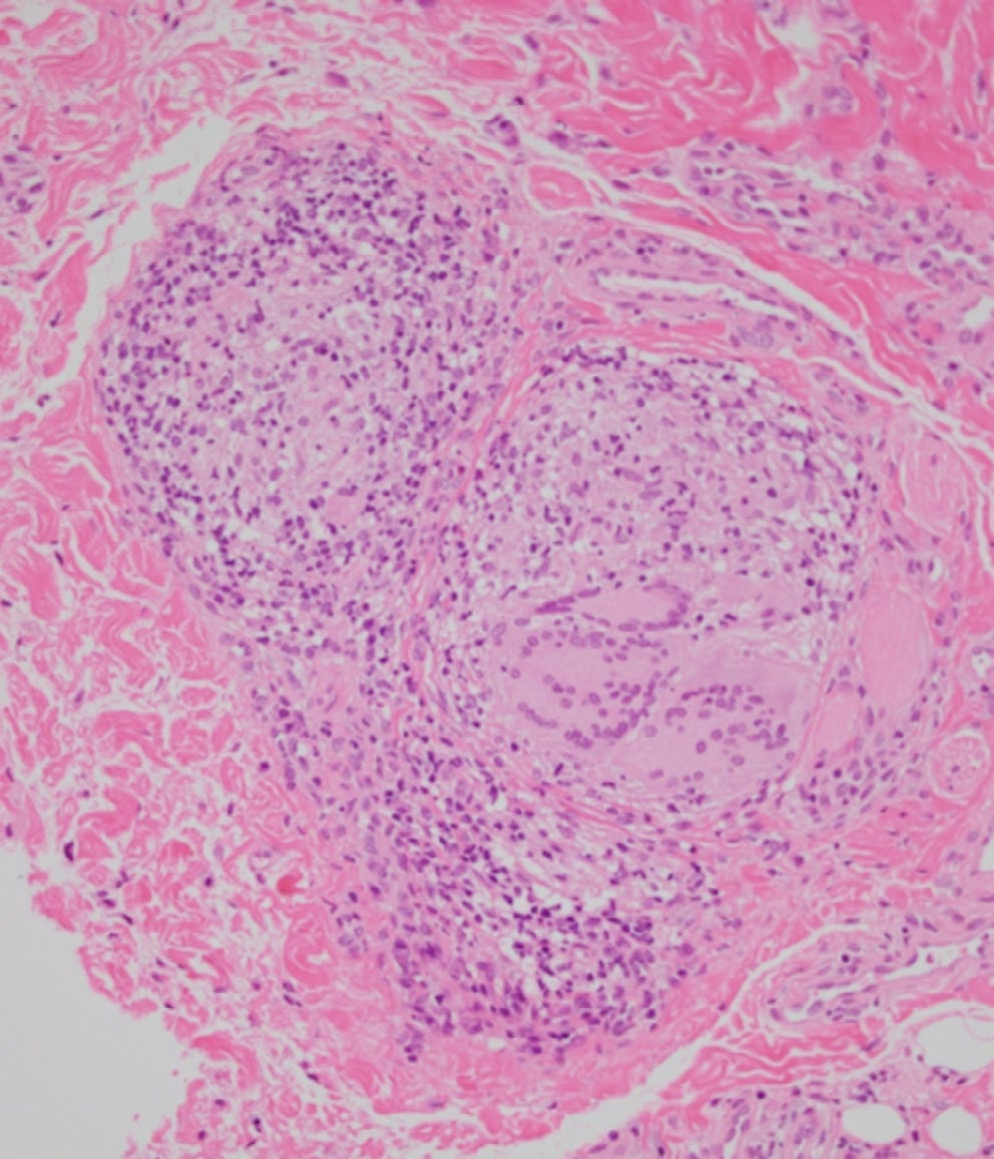
Histopathological examination of the upper lip showing granulomatous epithelioid cells surrounded by infiltrating reactive lymphocytes (hematoxylin and eosin stains; 200×).

**FIGURE 3:**
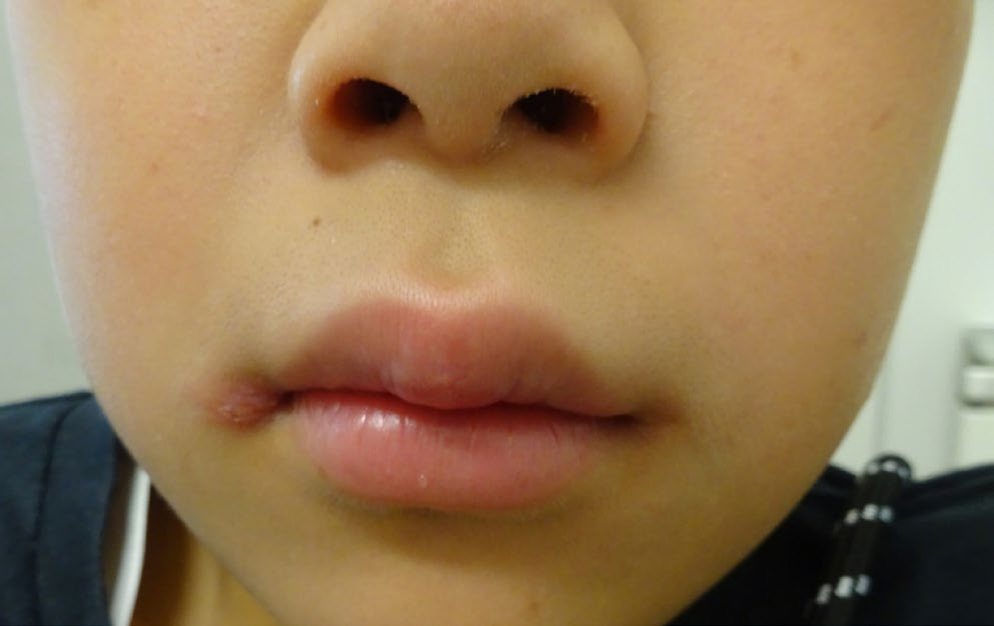
Posttreatment photograph: The painless swelling in the midline of the upper lip shows improvement to some extent, and the erosions have disappeared.

A healthy 10‐year‐old boy presented to our hospital with persistent swelling of the lips, more pronounced in the upper lip. The patient denied any associated fever, trauma, or gastrointestinal disturbances. He has no allergies, and both his medical and family histories were unremarkable. Angular cheilitis and painless lip swelling with erosion (Figure 1) were the only remarkable findings on physical examination without facial erythema and tongue plicata. Laboratory investigations, including complete blood count (hemoglobin 13.9 g/dL), liver and renal function tests (aspartate aminotransferase 23 U/L, alanine aminotransferase 11 U/L, albumin 4.6 g/dL), erythrocyte sedimentation rate (8 mm/h), CD4/CD8 ratio (2.0), serum C1 inhibitor (110%) and C4 levels (17 mg/dL), and fecal occult blood test results, were all within normal limits. Histopathological examination of the upper lip revealed parakeratotic hyperkeratosis with epidermal hyperplasia and epithelioid cell granuloma surrounded by infiltrating reactive lymphocytes (Figure 2). Microorganism tests, including Ziel‐Neelsen staining and mycobacterial culture, were negative. The diagnosis of cheilitis granulomatosa (CG) was made. The patient was initiated on topical betamethasone valerate and oral tranilast 300 mg daily, chosen for their low side effect profile. While swelling improved modestly, complete resolution was not achieved (Figure 3). We are closely monitoring the patient and considering intralesional corticosteroids and antibiotics as potential next treatment options.

## 2 Discussion

CG is a rare condition characterized by painless, recurrent swelling of one or both lips, with an estimated incidence of 0.08% in the general population and more infrequently in children [1]. The swelling is firm, nonpitting, and appears suddenly. While the exact etiology of CG remains elusive, it has been noted to be associated with conditions involving granuloma formation and chronic inflammation. The cause of erosions and angular cheilitis in this case is unclear but may involve chronic inflammation. It is also linked to Melkersson–Rosenthal syndrome, which includes swollen lips, facial nerve palsy, and a fissured tongue [2]. Importantly, CG may develop prior to gastrointestinal symptoms in some cases of Crohn's disease. Therefore, caution is warranted when initiating systemic immunomodulatory treatments, including systemic steroids, before thorough evaluation, such as a colonoscopy, for underlying chronic diseases.

Treating CG poses challenges, with no universally proven method, though corticosteroids remain central to treatment approaches for CG. Antibiotics such as minocycline and metronidazole, along with tranilast—a drug that inhibits the release of chemical mediators from mast cells—are used as additional treatments [3]. In fact, a combined regimen of intralesional corticosteroids, metronidazole, and minocycline has been reported to be successful in a child with CG [1].

When evaluating pediatric patients with CG, it is crucial to conduct a comprehensive workup before considering systemic immunomodulatory treatments. This includes a detailed medical history, physical examination, laboratory and microorganism tests, and colonoscopy.

## Author Contributions

**Shintaro Fujiwara:** conceptualization, data curation, writing – original draft. **Yousuke Higuchi:** conceptualization, supervision, writing – review and editing. **Yuma Nishimura:** data curation, writing – review and editing. **Sayuri Yokomizo:** data curation, writing – review and editing. **Yoko Shinno:** data curation, writing – review and editing.

## Ethics Statement

All procedures performed in this study were in accordance with the National Hospital Organization Okayama Medical Center's ethical standards and with the 1964 Helsinki Declaration and its later amendments.

## Consent

Written informed consent was obtained from the patient's parents to publish this case report and any accompanying images.

## Conflicts of Interest

The authors declare no conflicts of interest.

## Data Availability

The authors have nothing to report.
